# Functional Characterization of Arylalkylamine *N*-Acetyltransferase, a Pivotal Gene in Antioxidant Melatonin Biosynthesis from *Chlamydomonas reinhardtii*

**DOI:** 10.3390/antiox11081531

**Published:** 2022-08-05

**Authors:** Ok-Jin Hwang, Kyoungwhan Back

**Affiliations:** Department of Biotechnology, College of Agriculture and Life Sciences, Chonnam National University, Gwangju 61186, Korea

**Keywords:** brassinosteroids, cytokinin, green algae, melatonin, seed size, serotonin *N*-acetyltransferase, transgenic rice

## Abstract

Arylalkylamine *N*-acetyltransferase (AANAT) is a pivotal enzyme in melatonin biosynthesis that catalyzes the conversion of serotonin to *N*-acetylserotonin. Homologs of animal *AANAT* genes are present in animals, but not in plants. An *AANAT* homolog was found in *Chlamydomonas reinhardtii*, but not other green algae. The characteristics of *C. reinhardtii* *AANAT* (*CrAANAT*) are unclear. Here, full-length *CrAANAT* was chemically synthesized and expressed in *Escherichia coli*. Recombinant CrAANAT exhibited AANAT activity with a *K*_m_ of 247 μM and *V*_max_ of 325 pmol/min/mg protein with serotonin as the substrate. CrAANAT was localized to the cytoplasm in tobacco leaf cells. Transgenic rice plants overexpressing *CrAANAT* (*CrAANAT*-OE) exhibited increased melatonin production. *CrAANAT*-OE plants showed a longer seed length and larger second leaf angle than wild-type plants, indicative of the involvement of brassinosteroids (BRs). As expected, BR biosynthesis- and signaling-related genes such as *D2*, *DWARF4*, *DWARF11*, and *BZR1* were upregulated in *CrAANAT*-OE plants. Therefore, an increased endogenous melatonin level by ectopic overexpression of *CrAANAT* seems to be closely associated with BR biosynthesis, thereby influencing seed size.

## 1. Introduction

Arylalkylamine *N*-acetyltransferase (AANAT) is the penultimate enzyme for melatonin biosynthesis in animals and plants. It catalyzes the conversion of serotonin to *N*-acetylserotonin, which is the substrate for melatonin synthesis by *N*-acetylserotonin *O*-methyltransferase (ASMT) [1,2]. AANAT is also named serotonin *N*-acetyltransferase (SNAT); therefore, to differentiate them from animal *AANAT* genes, plant *AANAT* genes are frequently termed *SNAT* genes. Both animal AANAT and plant SNAT proteins belong to the GCN5-related *N*-acetyltransferase superfamily, which transfer an acetyl group from acetyl-coenzyme A (CoA) [3]. However, there is no significant amino sequence homology between animal AANATs and plant SNATs except for a few amino acids in the acetyl-CoA-binding domain [4]. Interestingly, an animal homolog of *arylalkylamine N-acetyltransferase* (*AANAT*) is present in the genome of *C. reinhardtii*, but not in other green algae or higher plants [3,5,6].

Based on the key role of AANAT (or SNAT) in melatonin biosynthesis, many animal *AANAT* and plant *SNAT* genes have been cloned and their recombinant proteins functionally characterized in vitro [7,8]. Ectopic overexpression of animal *AANAT* or plant *SNAT* genes in plant species resulted in increased melatonin synthesis [9,10]. The resulting *AANAT*- or *SNAT*-overexpressing transgenic plants exhibited increased melatonin synthesis and enhanced responses to biotic and abiotic stresses, including ultraviolet-B [11], high temperature [12], pathogen [13], salt [14], high light [15], cadmium [16,17], drought [18], oxidative stress [19], and cold exposure [20]. The enhanced tolerance to various stresses in *AANAT*- or *SNAT*-overexpressing plants was attributable to melatonin overproduction because melatonin not only has antioxidant activity but also induces antioxidant enzymes such as catalase, peroxidase, and superoxide dismutase [9,21].

*CrAANAT* was first characterized by Okazaki et al. [22]. CrAANAT transfers an acetyl group to serotonin. Transgenic tomato plants overexpressing *CrAANAT* had an increased melatonin level. However, the *K*_m_ and *V*_max_ values of recombinant CrAANAT and the phenotypes of *CrAANAT*-overexpressing transgenic plants are unknown. The aim of this work was to determine the enzyme kinetics of CrAANAT and its functional role in melatonin biosynthesis through heterologous expression in rice genome. We purified recombinant CrAANAT and determined the *K*_m_ and *V*_max_ values of its AANAT activity. Ectopic overexpression of *CrAANAT* in the rice genome increased the seed length and upregulated brassinosteroid (BR) (rather than cytokinin)-related gene expression.

## 2. Materials and Methods

### 2.1. Synthesis of C. reinhardtii AANAT

Based on AANAT of *C. reinhardtii* (CrAANAT; GenBank accession AB474787), the 192 codons of *CrAANAT* (including the stop codon) were manually optimized according to *SNAT2* codons of rice [23]. Codon-optimized synthetic *CrAANAT* was custom-synthesized by Bioneer (Daejeon, South Korea).

### 2.2. Affinity Purification of Various Recombinant C. reinhardtii AANAT Proteins from Escherichia coli Expression

Four different types of *Escherichia coli* vectors were employed to express the full-length synthetic *Chlamydomonas reinhardtii AANAT* (*CrAANAT*) DNA. Two vectors were pET300 (Invitrogen, Carlsbad, CA, USA) and pET28b (Novagen, San Diego, CA, USA) which are designed to express the CrAANAT in either N-terminal- or C-terminal- hexahistidine tagged form. The other two vectors were pET32b (Novagen) and pET60 (Novagen) which are designed to express the CrAANAT in N-terminal fusion proteins of either thioredoxin (Trx) or glutathione-s-transferase (GST). As for pET300, *CrAANAT-attB1* forward primer (5′-AAA AAG CAG GCT CCA TGG CTG AGG AGT CGC-3′) and *CrAANAT-attB2* reverse primer (5′-AGA AAG CTG GGT CTA GGC CTC AGC AGC CTC-3′) were used for PCR amplification with the synthetic *CrAANAT* gene followed by second PCR using adaptor primers with *attB* recombination sequences (*attB1* adaptor forward primer, 5′-GGG GAC AAG TTT GTA CAA AAA AGC AGG CT-3′; *attB2* adaptor reverse primer, 5′-GGG GAC CAC TTT GTA CAA GAA AGC TGG GT-3′). The resulting PCR product was gel purified and cloned into the pDONR221 Gateway^®^ vector (Invitrogen) via BP (between the *attB* and the *attP* sites) recombination. The pDONR221:CrAANAT gene entry vector was then recombined with the pET300 Gateway destination vector via LR (between the *attL* and the *attR* sites) recombination to form the pET300-CrAANAT vector. A pET28b-CrAANAT was constructed by PCR with *Nco*I forward primer (5′-ACC ATG GCT GAG GAG TCG CTC-3′) and *Xho*I reverse primer (5′-CTC GAG GGC CTC AGC AGC CTC TGC-3′). To generate pET60-CrAANAT, the *CrAANAT-attB1* forward primer and *6×His attB2* reverse primer (5′-AGA AAG CTG GGT TCA GTG GTG GTG GTG GTG-3′) were used to amplify the *CrAANAT* with the pET28b-CrAANAT plasmid as a template. The resulting PCR product was further amplified with the *attB1* adaptor forward and *attB2* adaptor reverse primers followed by BP and LR recombination reactions as described above. A pET32b-CrAANAT was constructed by ligating the *Nco*I and *Xho*I insert prepared during the pET28b-CrAANAT vector construction. All plasmids were transformed into *E. coli* strain BL21(DE3) (Novagen).

### 2.3. Purification of Recombinant CrAANAT Proteins

Each 10 mL of *E. coli* overnight culture of *E. coli* containing pET300-CrAANAT, pET28b-CrAANAT, pET60-CrAANAT, and pET32b-CrAANAT plasmid vectors was inoculated into 100 mL of Terrific Broth (20 g/L Bacto-tryptone, 24 g/L Bacto-yeast extract, 4 mL/L glycerol, and phosphate buffer [0.017 M monopotassium phosphate and 0.072 M dipotassium phosphate]) containing with 50 mg/L ampicillin or 50 mg/L kanamycin (pET28b-CrAANAT) and incubated at 37 °C until the optical density at 600 nm reached 1.0 about 3 to 4 h. The culture was added with 1 mM isopropyl-*β*-D-thiogalactopyranoside (Sigma, St. Louis, MO, USA) and grown at 28 °C with shaking at 180 rpm for 5 h. The protein was purified via affinity nickel ion chromatography according to the column manufacturer’s instructions (Qiagen, Tokyo, Japan).

### 2.4. Measurement of Serotonin N-Acetyltransferase (SNAT) Enzyme Activity

Two types of purified recombinant CrAANAT proteins were incubated in 100 μL of 100 mM potassium phosphate (pH 8.8 or various pH values) in the presence of 0.5 mM serotonin and 0.5 mM acetyl-coenzyme A. SNAT enzyme assays were conducted at 45 °C for 30 min (or various temperatures) and stopped by adding 25 μL of methanol. Then, 10 μL aliquots of the reaction mixture were subjected to high-performance liquid chromatography (HPLC) coupled to a fluorescence detector system to detect *N*-acetylserotonin as described previously [24]. Non-enzymatic reaction products that were generated without the CrAANAT enzymes were deducted. To acquire substrate affinity (*K*_m_) and maximum reaction rate (*V*_max_), various substrates (50 to 2000 μM serotonin) and enzyme concentrations (0.2 to 1 μg) were employed. The *K*_m_ and *V*_max_ values were calculated using Lineweaver–Burk plots. Protein concentration was determined using Bradford assays (Bio-Rad, Hercules, CA, USA). The analyses were performed in triplicate.

### 2.5. Subcellular Localization of CrAANAT

The pER-mCherry vector which was kindly donated by Dr. H. Kang (Texas State University, San Marcos, TX, USA) was used for assessing the localization of CrAANAT protein in tobacco leaves. Full-length *CrAANAT* cDNA was amplified by PCR with two *Asc*I containing primers (*CrAANAT Asc*I forward primer 5′-GGC GCG CCA TGG CTG AGG AGT CGC TCG-3′; *CrAANAT Asc*I reverse primer 5′-GGC GCG CCG GGC CTC AGC AGC CTC TGC-3′). The resulting PCR product was first cloned into the T&A cloning vector (T&A:CrAANAT; RBC Bioscience, New Taipei City, Taiwan) from which the *Asc*I insert of CrAANAT was produced and cloned into the binary pER8-mCherry vector at the *Asc*I restriction sites downstream of the estrogen-inducible XVE promoter to generate CrAANAT-mCherry fusion proteins. The plasmid was transformed into *Agrobacterium tumefaciens* strain GV2260 using the freeze-thaw method. As for a transient expression analysis of CrAANAT-mCherry, the leaves of two-week-old tobacco (*Nicotiana benthamiana*) plant, a native Australian species, were infiltrated with *A. tumefaciens* strain GV2260 carrying pER8:CrAANAT-mCherry plasmid. The transformed tobacco leaves were then examined using confocal microscopy to determine the subcellular localization of the CrAANAT-mCherry fusion proteins. Further treatment with β-estradiol (Sigma Aldrich, St. Louis, MO, USA) and confocal microscopy analysis were described previously [24].

### 2.6. Vector Construction and Production of CrAANAT-Overexpressing Transgenic Rice Plants

The pDONR221:CrAANAT gene entry vector harboring the synthetic *CrAANAT* gene was then recombined with the pIPKb002 destination vector [25] via LR recombination to yield pIPKb002-CrAANAT, which was transformed into *Agrobacterium tumefaciens* strain LBA4404. We used *Agrobacterium*-mediated rice transformation with the coculture with rice scutelum-derived calli to generate transgenic rice (*Oryza sativa* cv. Dongjin, a Korean *japonica* cultivar) plants as described previously [26].

### 2.7. Plant Growth Conditions

Rice (*Oryza sativa* cv. Dongjin, a Korean *japonica* cultivar) seeds of both wild type and *CrAANAT* overexpression (*CrAANAT*-OE) were sterilized with 2% sodium hyphochlorite and rinsed with sterile distilled water. Sterilized seeds were grown on half-strength Murashige and Skoog (MS) medium under cool daylight fluorescent lamps (60 μmol m^−2^ s^−1^) (Philips, Amsterdam, Netherlands) in 14-h light/10-h dark photoperiod at 28 °C/24 °C (day/night) for 7 days. Germinated seeds were grown in a paddy field at the Chonnam National University (35°09′ N and 126°54′ W; 53 m a.s.l), Gwangju, Korea in 2021. The distance between the rice plants within a row was 30 cm, and the distance between the rows was 30 cm. Grain length, grain width and 1000-grain weight were measured after harvesting followed by drying for 1 month at room temperature of about 26 °C.

### 2.8. Chemical Treatment

Seven-day-old rice seedlings were incubated in 30 mL of 100 μM 5-methoxytrytamine (Sigma-Aldrich, St. Louis, MO, USA) dissolved in 0.02% ethanol for 1 day under cool daylight fluorescent lamps (60 μmol m^−2^ s^−1^) (Philips) in 14-h light/10-h dark photoperiod at 28 °C/24 °C (day/night). The 0.02% ethanol was used as a control. The leaves and stems were harvested for further analyses.

### 2.9. Quantitative Real Time-Polymerase Chain Reaction (qRT-PCR) Analysis

Total RNA from rice plants was isolated using a NucleoSpin RNA Plant Kit (Macherey-Nagel, Düren, Germany). First-strand cDNA was synthesized from 2 μg of total RNA using EcoDry^TM^ Premix (Takara Bio USA, Inc., Mountain View, CA, USA). qRT-PCR was performed in a Mic qPCR Cycler system (Biomolecular Systems, Queensland, VIC, Australia) with specific primers and the TB Green^®^ Premix Ex Taq^TM^ (Takara Bio Inc., Kusatsu, Shiga, Japan). The expression of genes was analyzed using Mic’s RQ software v2.2 (Biomolecular Systems) and normalized to *actin 1* (*ACT1*). Reverse transcription (RT)-PCR and quantitative real-time (qRT)-PCR were performed with the primer set (Table S1).

### 2.10. Quantification of Melatonin

Frozen samples (0.1 g) were ground in liquid nitrogen with the use of the TissueLyser II (Qiagen, Tokyo, Japan) and extracted with 1 mL of chloroform. The chloroform extracts were centrifuged for 10 min at 12,000× *g*, and the supernatants (200 μL) were completely evaporated and dissolved in 0.1 mL of 40% methanol, and 20-μL aliquots were subjected to HPLC using a fluorescence detector system (Waters, Milford, MA, USA) as described previously [27]. In brief, melatonin was detected at 280 nm (excitation) and 348 nm (emission) on a Sunfire C18 column (Waters 4.6 × 150 mm) in the following gradient elution condition: from 42% to 50% methanol in 0.1% formic acid for 27 min, followed by isocratic elution with 50% methanol in 0.1% formic acid for 18 min at a flow rate of 0.15 mL/min. All measurements were performed in triplicate.

### 2.11. Statistical Analyses

The data were analyzed using analysis of variance (ANOVA) using IBM SPSS Statistics 23 software (IBM Corp., Armonk, NY, USA). Means with asterisks indicate significantly different values at *p* < 0.05, according to a Fisher’s least significant difference (LSD) test. All data are presented as mean ± standard deviations.

## 3. Results

### 3.1. Codon-Optimized Synthesis of CrAANAT and Its Expression in Escherichia coli

**Figure 1:**
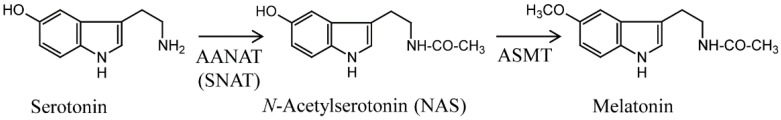
Schematic diagram of melatonin biosynthesis. AANAT is also known as serotonin *N*-acetyltransferase (SNAT) in plants. ASMT, *N*-acetylserotonin *O*-methyltransferase.

**Figure 2:**
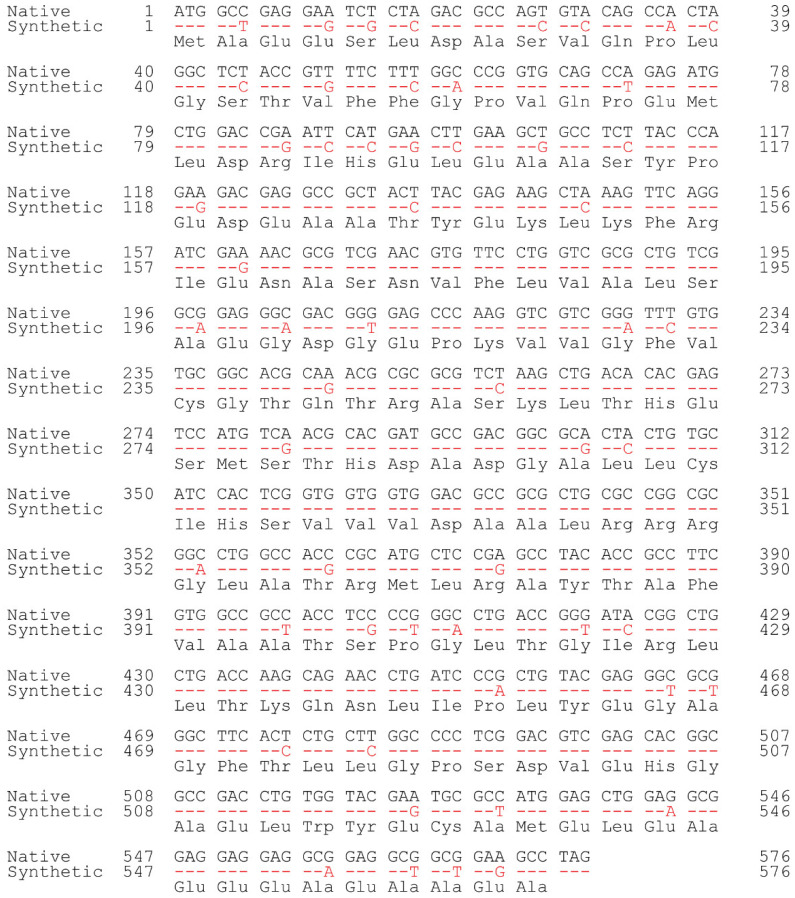
Nucleotide sequence alignments of native (black writing; AB474787) and synthetic *CrAANAT* (red writing) genes. Identity is denoted by red dashes. The nucleotide sequence of synthetic *CrAANAT* was codon-optimized with reference to rice *SNAT2* (AK068156).

The full-length *CrAANAT* nucleotide sequence (encoding 191 amino acids) was chemically synthesized based on the codon usage of rice *SNAT2* (AK068156) exhibiting a 70% G+C content. Among the 192 codons of *CrAANAT*, 55 were modified in the synthetic *CrAANAT* gene, increasing the G+C content from 64% to 67% (Figure 1 and Figure 2).

**Figure 3:**
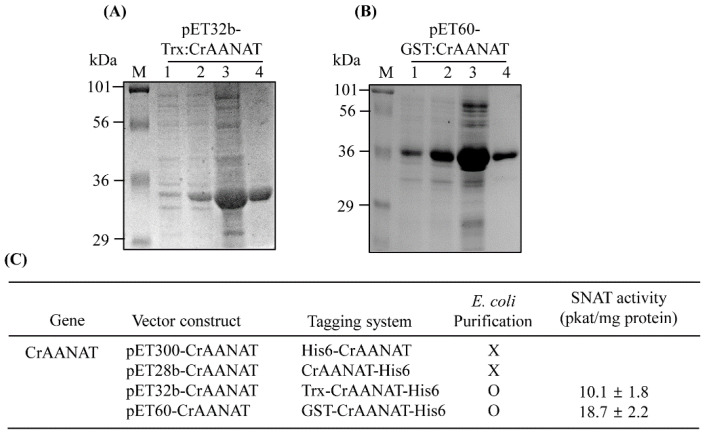
Expression of *CrAANATs* in *Escherichia coli*. (**A**) Affinity purification of thioredoxin (Trx)-CrAANAT. (**B**) Affinity purification of glutathione-s-transferase (GST)-CrAANAT. (**C**) SNAT activity of purified recombinant CrAANAT. Protein samples were separated by SDS-PAGE and stained with Coomassie brilliant blue. M, molecular size standard; 1, total proteins in 10 μL aliquots of bacterial cell culture without isopropyl *β*-D-thiogalactopyranoside (IPTG); 2, total proteins after IPTG treatment; 3, 20 μg of soluble protein extract from supernatant after centrifugation at 15,000× *g*; 4, recombinant proteins purified by affinity (Ni-NTA) chromatography. X, no purification; O, successful purification.

Synthetic *CrAANAT* was expressed in *E. coli* as a fusion protein with an N- or C-terminal hexa-histidine (His6) tag, followed by Ni^2+^ affinity purification (Figure 3). Intact His6-CrAANAT and CrAANAT-His6 were insoluble and so could not be purified using a Ni^2+^ affinity column. However, CrAANAT fusions with thioredoxin (Trx) or glutathione-s-transferase (GST) were soluble and subjected to Ni^2+^ affinity purification (Figure 3A,B). Recombinant Trx-CrAANAT-His6 exhibited serotonin *N*-acetyltransferase (SNAT) activity of 10.1 pkat/mg protein at 45 °C and pH 8.8, compared to 18.7 pkat/mg protein for GST-CrAANAT-His6 (Figure 3C). Therefore, CrAANAT transfers an acetyl group from acetyl-CoA to serotonin to produce *N*-acetylserotonin, the final substrate in melatonin biosynthesis [4].

### 3.2. Kinetics of Recombinant CrAANAT

**Figure 4:**
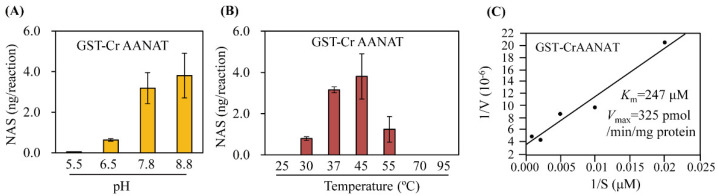
Conversion of serotonin to *N*-acetylserotonin as a function of (**A**) pH and (**B**) temperature. (**C**) Substrate affinity (*K*_m_) and maximum reaction rate (*V*_max_) values of CrAANAT. Purified recombinant GST-CrAANAT (1 μg) was incubated at a range of pH values and temperatures for 30 min. *K*_m_ and *V*_max_ values were determined using Lineweaver-Burk plots. *N*-Acetylserotonin, an in vitro enzymatic product, was quantified by high-performance liquid chromatography. The data are means ± standard deviation (n = 3).

SNAT activity peaked at pH 8.8 and was similar at pH 7.8 (Figure 4). This high pH optimum is consistent with that of other plant SNAT proteins [8,13,23,28,29] but unlike animal AANAT proteins (pH 6.7) [30,31]. SNAT activity was fourfold lower at pH 6.5 than at pH 8.8. SNAT activity was highest at 45 °C followed by 37 °C. The high level of SNAT activity at 37 °C is similar to other animal AANAT proteins but not plant SNAT proteins [8,30,31]. The *K*_m_ and *V*_max_ values were 247 μM and 5.4 pkat/mg protein (325 pmol/min/mg protein), respectively. The *K*_m_ value of CrAANAT is similar to those of rice [24], Arabidopsis [29], and tobacco [8], but lower than that of red algae [32] and cyanobacteria [33]. The *K*_m_ value of CrAANAT was threefold higher than that of sheep AANAT [7]. In contrast, the *V*_max_ value of CrAANAT was lower than that of plant SNATs and sheep AANATs. Therefore, the kinetics of CrAANAT has similarities to those of plant SNATs and animal AANATs. To be more precise, the CrAANAT is close to plant SNAT at the level of *K*_m_ value, but to animal AANAT at the level of *V*_max_ value.

### 3.3. Subcellular Localization of CrAANAT

**Figure 5:**
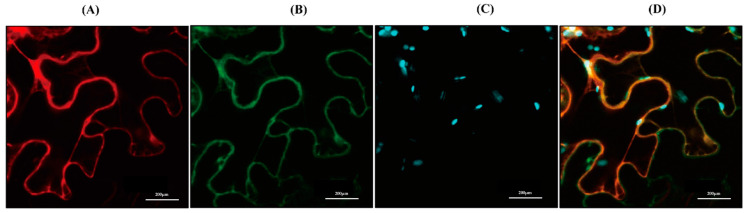
Localization of CrAANAT. (**A**) Red fluorescence of CrAANAT-mCherry. (**B**) Green fluorescence of cytoplasmic GFP. (**C**) Cyan fluorescence of chloroplasts. (**D**) Merged image (A + B + C). Leaves of 30-day-old tobacco (*Nicotiana benthamiana*) seedlings were infiltrated with *Agrobacterium* (GV2260) containing XVE-inducible CrAANAT-mCherry, or constitutive 35S:GFP (cytosolic marker). Bars, 20 μm. Synthetic *CrAANAT* was used in place of native *CrAANAT* (AB474787).

Because in silico TargetP analysis of CrAANAT showed no transit or signal sequence [34], we hypothesized that CrAANAT is cytoplasmic. We subcloned CrAANAT into a binary vector to express the CrAANAT-mCherry fusion protein under the control of the estrogen-inducible XVE promoter. *Agrobacterium* cells harboring the binary vector were infiltrated into tobacco leaves followed by transgene induction by β-estradiol. Confocal microscopy showed that CrAANAT-mCherry exhibited strong mCherry fluorescence, which co-localized with the green fluorescence of cytoplasmic GFP (Figure 5). Therefore, CrAANAT localizes to the cytoplasm, as does sheep AANAT [35]. CrAANAT in the cytoplasm is unlike the chloroplastic localizations of other plant SNAT proteins [8,13,24].

### 3.4. Transgenic Rice Plants Overexpressing CrAANAT (CrAANAT-OE)

**Figure 6:**
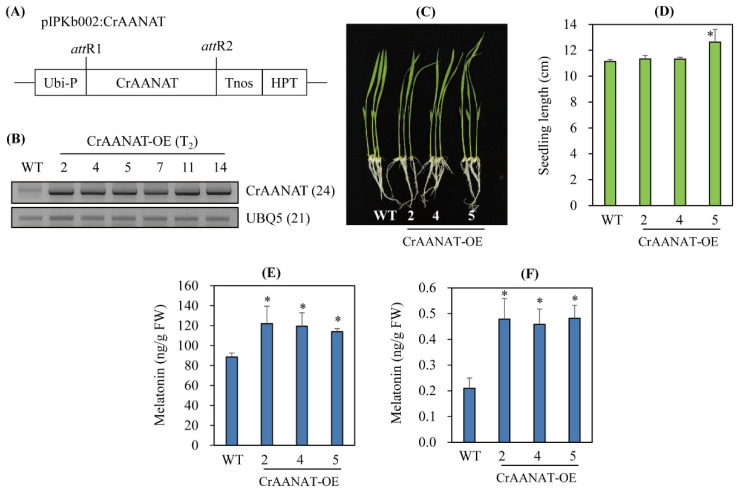
Structure of the pIPKb002-CrAANAT binary vector and its overexpression in transgenic rice plants. (**A**) Schematic diagram of the pIPKb002:CrAANAT binary vector. (**B**) Expression of *CrAANAT* in wild type (WT) and *CrAANAT*-overexpressing (*CrAANAT*-OE) seedlings (T_2_). (**C**) Phenotypes of 7-day-old rice seedlings of WT and *CrAANAT*-OE transgenic plants (T_2_). (**D**) Seedling lengths of WT and *CrAANAT*-OE plants. (**E**) Melatonin contents in 7-day-old rice seedlings after 100 μM 5-methoxytryptamine (5-MT) in 0.02% ethanol. (**F**) Melatonin contents in 7-day-old rice seedlings after 0.02% ethanol treatment. Seven-day-old rice seedlings were rhizospherically challenged with 100 μM 5-MT for 24 h in 0.02% ethanol. *, significant difference from wild type (*p* < 0.05; ANOVA, followed by Fisher’s LSD test). The numbers of PCR cycles are shown in parentheses. Synthetic *CrAANAT* was overexpressed in the rice genome. *Ubi-P*, maize ubiquitin promoter; *HPT*, hygromycin phosphotransferase; *Tnos*, nopaline synthase terminator. GenBank accession number of *UBQ5*, Os03g13170.

To determine whether *CrAANAT* is functionally coupled to melatonin biosynthesis in vivo, we generated *CrAANAT*-OE under the control of the maize ubiquitin promoter (Figure 6A).

Six homozygous T_2_ *CrAANAT*-OE were selected from 11 independent T_1_ transgenic rice seeds (Figure 6A,B). These T_2_ *CrAANAT*-OE showed *CrAANAT* overexpression whereas *CrAANAT* transcript was not detected in wild type (WT). The T_2_ homozygous *CrAANAT*-OE, particularly line 7, showed slightly increased seedling growth (Figure 6C,D). When these T_2_ homozygous transgenic seedlings were rhizospherically challenged for 24 h with 100 μM 5-methoxytryptamine (5-MT), a substrate for AANAT-catalyzed melatonin biosynthesis, the WT and *CrAANAT*-OE lines produced 83 and 116 ng/g fresh weight (FW) (Figure 6E). Therefore, CrAANAT converts 5-MT into melatonin by acetylating 5-MT, as do most animal AANAT and plant SNAT proteins [8,36]. In the absence of 5-MT, the WT and *CrAANAT*-OE lines produced melatonin at 0.2 and 0.45 ng/g FW (Figure 6F). Therefore, ectopic overexpression of *CrAANAT* increased AANAT activity compared to wild type. *CrAANAT* overexpression was functionally associated with enhanced production of melatonin in the *CrAANAT*-OE line compared to wild type.

**Figure 7:**
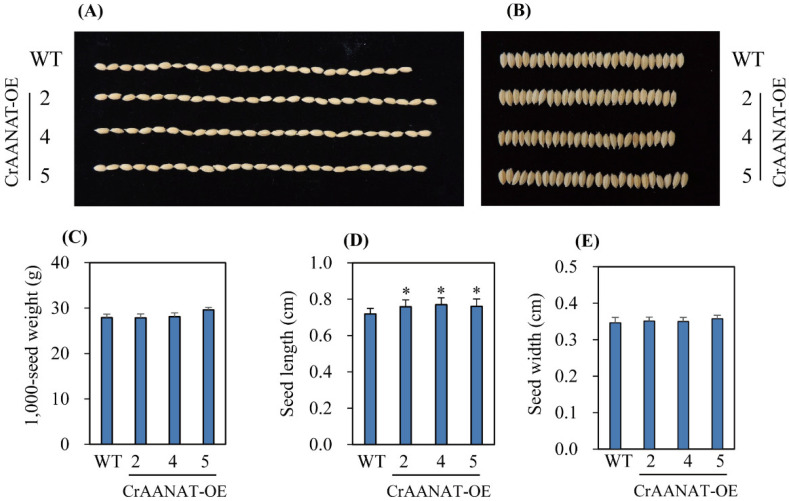
Morphology of T_2_ homozygous transgenic *CrAANAT*-OE seeds. (**A**) Photograph of WT and *CrAANAT*-OE seeds (T_2_). (**B**) Photograph of WT and *CrAANAT*-OE seeds (T_2_). (**C**) The weight of 1000 seeds of WT and *CrAANAT*-OE transgenic rice (T_2_). (**D**) Seed length. (**E**) Seed width. * Significant difference from wild type (*p* < 0.05; Fisher’s LSD test).

**Figure 8:**
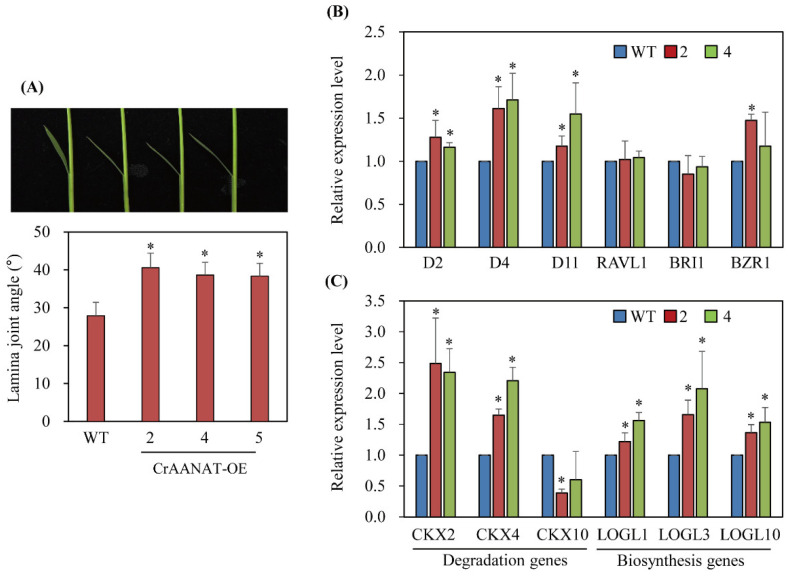
Second-leaf angle and cytokinin and brassinosteroid (BR) biosynthesis-related gene expression. (**A**) Second-leaf angle. (**B**) Quantitative real-time (qRT)-PCR of BR biosynthesis- and signaling-related genes. (**C**) qRT-PCR of cytokinin biosynthesis- and degradation-related genes. Fourteen-day-old rice seedlings grown in soil were used to measure the lamina angle of the second leaf; meristem parts of rice seedlings, including second leaves, were subjected to total RNA extraction and qRT-PCR. GenBank accession numbers are *CKX2, cytokinin oxidase2* (Os01g0197700); *CKX4* (Os01g0940000); *CKX10* (Os06g0572300); *LOGL1, LONELY GUY LIKE phosphoribohydrolase1* (Os01g0708500); *LOGL3* (Os03g0109300); *LOGL10* (Os10g0479500); *D2, DWARF2* (XP-015611433); *D4, DWARF4* (AB206579); *D11, DWARF11* (AK106528); *RAVL1, RAV Like1* (Os04g0581400); *BRI1* (AK101085); *BZR1* (Os07g39220); *ACT1* (Os03g50885). * Significant difference from wild type (*p* < 0.05; Fisher’s LSD test).

Homozygous T_2_ *CrAANAT*-OE seeds were of greater length, but lesser width, than WT seeds (Figure 7). The 1000-seed weight was similar in the *CrAANAT*-OE lines and wild type. To determine whether the increase in seed length is associated with BRs, we measured the second leaf angle, a phenotypic marker of BR levels. The second-leaf angle was larger in the *CrAANAT*-OE seedlings than in WT seedlings (Figure 8A), indicating increased BR levels. Cytokinins also regulate seed size in plants [37]. To identify the hormones responsible for the increased seed length in the *CrAANAT*-OE lines, we evaluated the expression levels of BR- and cytokinin-related genes. The expression levels of BR-related genes were significantly increased in the *CrAANAT*-OE lines (the BR biosynthesis-related genes *DWARF* [*D*]*2*, *D4*, and *D11* and *BRASSINOZOLE-RESISTANT 1* (*BZR1*), encoding a transcription factor that regulates BR-responsive gene expression; Figure 8B) [38,39]. By contrast, cytokinin degradation genes (*CKX2*, *CKX4*, and *CKX10*) and cytokinin biosynthesis genes (*LOGL1*, *LOGL3*, and *LOGL10*) were upregulated. Therefore, the increased lamina angle and seed length in the *CrAANAT*-OE line compared to wild type are caused by BRs rather than cytokinins.

## 4. Discussion

The final two genes in melatonin biosynthesis are *AANAT* (or *SNAT*) and *ASMT* [4]. *AANAT* was first cloned and characterized in sheep and rats [40,41]. *AANAT* homologs have been cloned from fish [42], humans [31], yeast [43], and mosquitos [44]. AANAT plays a rate-limiting role in melatonin biosynthesis by acetylating serotonin and 5-MT, thus synthesizing *N*-acetylserotonin and melatonin, respectively, in animals and plants [8,36]. An animal *AANAT* homolog was reported in the green alga *C. reinhardtii*, but not in other green algae or higher plants [5]. Okazaki et al. [22] expressed *C. reinhardtii AANAT* in *E. coli* and reported that the purified GST-CrAANAT fusion protein transferred an acetyl-CoA group to serotonin but did not determine its *K*_m_ and *V*_max_ values. Furthermore, its overexpression in tomato increased melatonin synthesis, but this did not affect the plant phenotype. The *K*_m_ and *V*_max_ values of purified recombinant CrAANAT for serotonin indicated that *CrAANAT* encodes a SNAT enzyme. Similar to other plant SNAT proteins, CrAANAT prefers a high pH, but an optimum temperature of around 37 °C, like animal AANATs [45]. This optimum temperature of CrAANAT is in contrast to that of other plant SNAT enzymes (45 °C to 55 °C) [8], indicating that CrAANAT possesses characteristics of animal AANAT and plant SNAT proteins.

**Figure 9:**
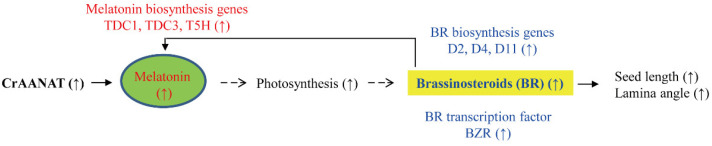
Proposed model for the melatonin-mediated increase in seed length. The increase in melatonin synthesis caused by ectopic overexpression of *CrAANAT* leads to an increased seed length and leaf angle, key phenotypic markers of increased BR levels in rice. A number of BR biosynthesis- and signaling-related genes were upregulated in the *CrAANAT*-overexpressing rice plants. These data suggest that melatonin is positively associated with BR levels in rice plants. The melatonin-induced BR increase is an indirect effect on photosynthesis—melatonin is positively coupled to starch synthesis and photosynthesis, which affect BR biosynthesis [57]. BR triggers melatonin biosynthesis by inducing the expression of melatonin biosynthesis genes such as *tryptophan decarboxylase 1* (*TDC1*), *TDC3*, and *tryptamine 5-hydroxylase* (*T5H*) [64]. Solid arrows, confirmed functions; dashed arrows, steps not yet demonstrated in rice. ↑, upregulation.

Melatonin has diverse functions in plant growth, development, and biotic and abiotic stress responses [9,10,46,47,48,49] by modulating the cellular redox balance [50,51,52] and protein quality control [53]. Additionally, melatonin functions in concert with other plant hormones during growth and under stressful conditions [54,55]. Melatonin directly influences hormone levels in *Arabidopsis thaliana*, in which exogenous melatonin promotes primary root growth via the indole-3-acetic acid signaling pathway [56]. Indirect effects of melatonin on plant hormones have been reported in plants with up- or down-regulated melatonin synthesis [57,58]. Melatonin did not directly enhance gibberellic acid (GA) synthesis [57] and BR effects such as leaf angle increases [58]. However, melatonin suppression in an *A. thaliana* knockout mutant (*snat1*) and *SNAT2* RNAi rice plants resulted in decreased levels of GA and BR, respectively. This was caused by decreased starch synthesis, which is promoted by melatonin [57,59,60]. An increased endogenous melatonin level in transgenic rice overexpressing caffeic acid *O*-methyltransferase (COMT) markedly increased the seed size and rice yield as a result of elevated cytokinin levels [61]. Transgenic rice plants overexpressing *SNAT* genes from Archaea and rice showed an increase in seed size [62,63]; however, whether this is caused by cytokinins or BRs is unclear. Here, the increased rice seed length caused by an increased endogenous melatonin level was linked to increased BR levels due to upregulation of BR biosynthetic genes (Figure 9), not increased cytokinin levels as in *COMT*-overexpressing rice [61]. Moreover, the increase in BR levels caused by endogenous melatonin overproduction induces melatonin biosynthesis by activating the expression of melatonin biosynthetic genes such as *tryptophan decarboxylase 1* (*TDC1*), *TDC3*, and *tryptamine 5-hydroxylase* (*T5H*) [64] (Figure 9). In sum, the effect of melatonin on seed size and yield [17,61] and stress tolerance [65,66] suggests that the generation of melatonin-rich rice plants by exogenous application [64] or transgenic approaches [4] would increase yields and resistance to many biotic and abiotic stresses and enable the production of melatonin-rich foods with health benefits [67].

## 5. Conclusions

CrAANAT had homology to animal AANAT proteins but not to plant SNAT proteins. Its optimum temperature was 37 °C, similar to animal AANAT proteins, but its optimum pH was pH 8.8, similar to plant SNAT proteins, demonstrating that CrAANAT has characteristics of both animal AANAT and plant SNAT proteins. Ectopic overexpression of *CrAANAT* in the rice genome led to an increase in melatonin content, leaf angle, and seed length, indicative of enhanced BR biosynthesis. An increased BR level in *CrAANAT*-OE rice plants was indirectly verified by the upregulation of BR biosynthetic genes such as *D2*, *D4*, and *D11*. This is the first report of an increase in BR biosynthesis by the ectopic overexpression of *AANAT* or *SNAT* in transgenic plants. Many RNAi transgenic rice plants with downregulated melatonin synthesis show decreased BR levels and leaf angle, suggesting a close relationship between melatonin and BR levels in rice plants [39,61]. The *CrAANAT* can be used as a source gene for a simultaneous increase of melatonin and BR which will lead to improved plant growth and stress tolerance conferred by either melatonin or BR alone or a combination of both [38,39,50].

## Data Availability

Data presented in this study are available within the article.

## Supplementary Materials

The following supporting information can be downloaded at: https://www.mdpi.com/article/10.3390/antiox11081531/s1, Table S1: Sequences of primers used for polymerase chain reaction.
