# Supplementary material for: Functional Characterization of Arylalkylamine N-Acetyltransferase, a Pivotal Gene in Antioxidant Melatonin Biosynthesis from Chlamydomonas reinhardtii
Source: Antioxidants (Basel). 2022 Aug 5;11(8):1531. doi: 10.3390/antiox11081531 (PMC9405056; doi:10.3390/antiox11081531)
Supplement: Supplementary file 1 [file antioxidants-11-01531-s001.zip › antioxidants-1838230-supplementary.pdf]

Table S1. Sequences of primers used for polymerase chain reaction.

Gene	Forward (5´-3´)	Reverse (5´-3´)
CrAANAT	ATG GCT GAG GAG TCG CTC G	GGC CTC AGC AGC CTC TGC
UBQ5	CCG ACT ACA ACA TCC AGA AGG AG	AAC AGG AGC CTA CGC CTA AGC
OsLOGL1	GCA GAA GGA TTT ATT AAG GCC G	TTC CAT AAC CAA GCT CTG ACA T
OsLOGL3	TGA ACG TTG ATG GGT ACT ACA A	ATC ATG GTA AGG GAC GTA TTC C
OsLOGL10	GAA GAA GAG GAG CTA CCA TGA C	TCA TGA GAG TCT TGG GAA TGA C
OsCKX2	GAG GTG TTC TAC ACG GTG GG	GCA GGT ACT GCT TGT AGG CT
OsCKX4	GCA CCA TCA TCC TCA GGT CA	AAG TGG GCT TTC CAC TGC TT
OsCKX10	GCT TCC GTG GAT CAG AAA CTT G	ATG CGT CCC TGA CGA ACA
D2	ATG TGA TAA CAG AGA CGC TGC GGT	TGG TGA CCA AGT GGT GAA GGA AGA
D4	GGA GAA GAA CAT GGA ATC AC	GTA ATC TTG AAC GCG GAT ATG
D11	TGA GGC ACT GAG ATG TGG	AAG GTG ATG GAG GAA GAA
RAVL1	CGA CTT CCG CAA CAT CAA	GGC ATC CGT AGC GAC AAT
BRI1	CAG CTA CTT GGC TAT CTT GAA GCT CAG C	CCA TTC TTG TTG AAG GTG TAC TCC GTG C
BZR1	ATG ACG GCC ATT ATT GCC GAG CA	TCG CCC AAA TCG CAG CAT
ACT1	TGC TAT GTA CGT CGC CAT CCA	AAT GAG TAA CCA CGC TCC GTC AA
